# How residential environment governance improves residents’ health and well-being—evidence from a quasi-natural experiment

**DOI:** 10.3389/fpubh.2026.1851697

**Published:** 2026-07-02

**Authors:** Yangyang Xu, Jialin Liu, Mengyu Wang

**Affiliations:** 1 School of Economics, Lanzhou University of Finance and Economics, Lanzhou, China; 2 International Exchange Center, Lanzhou University of Finance and Economics, Lanzhou, China; 3 School of Statistics, Shandong Technology and Business University, Yantai, China

**Keywords:** ecological restoration and urban renovation, medical expenditure, quasi-natural experiment, residential environment, residents’ health and well-being

## Abstract

**Introduction:**

Improvement in urban-rural residential environment is not only vital to national health and well-being, but also an important pillar for safeguarding global public health security and promoting the sustainable development of human health.

**Methods:**

Taking China’s pilot program of the Ecological Restoration and Urban Renovation (ERUR) as a quasi-natural experiment, this study matches five waves of the China Health and Retirement Longitudinal Study (CHARLS) dataset from 2011 to 2020, constructs a difference-in-differences (DID) model to examine the impact and channel of residential environment governance on residents’ health and well-being.

**Results:**

The results show that residential environment governance significantly improves residents’ health status, and this effect is more pronounced among urban residents, individuals with low education levels, and those living in highly livable regions and areas with inadequate medical resources. Channel analysis reveals that residential environment governance enhances residents’ health through three channels: ecological environment restoration, infrastructure upgrading and health awareness improvement. Further analysis indicates that residential environment governance does not directly reduce medical expenditure burden; instead, it moderately increases residents’ utilization of medical services and self-medication expenditure. This phenomenon essentially reflects the improved awareness of health management and the optimized structure of health demand, rather than an irrational expansion of overall medical demand.

**Discussion:**

The research findings provide a pragmatic foundation for establishing a developmental framework that fosters reciprocal enhancement between environmental sustainability and public health.

## 1 Introduction

Residents’ health serves not only as a fundamental prerequisite for individual well-being but also as a crucial indicator of national social welfare and socioeconomic development. In recent years, as living standards and health awareness continue to rise, residential environment governance has attracted increasing attention from academics and policymakers. While health challenges are prevalent globally, China faces particularly severe population health pressures amid rapid socioeconomic and demographic transitions. From a physical health perspective, China is witnessing a trend of chronic diseases manifesting at younger ages. In 2024, the adult hypertension prevalence rate reached as high as 31.60%, affecting over 245 million individuals. Meanwhile, the number of older adults with activity limitations stood at 46.54 million. Regarding mental health, prevalence rates for common psychological issues such as cognitive impairment and depression show a consistent annual increase. The lifetime prevalence of depressive disorders among Chinese adults is approximately 6.80% (1), indicating substantial room for improvement in overall health literacy. Against this background, systematically enhancing resident health outcomes and bolstering the foundation for high-quality population development have become an urgent public policy concern.

The impact of the residential environment on individual health exhibits long-term and progressive characteristics, making the creation of health-friendly ecological and urban environments indispensable for improving residents’ health outcomes (2). On one hand, ecological restoration, through measures such as hillside rehabilitation, vegetation recovery, water management, pollution reduction, and green space expansion, can create a more livable natural environment for residents (3). On the other hand, urban environmental renovation carries out multiple initiatives. These include expanding public spaces for residents’ daily activities, renovating old buildings, building sports and medical facilities, and improving older adults care and medical service systems. Such improvements deliver accessible amenities and services, and ultimately promote residents’ health and well-being (4).

Existing research on residential environment governance effect can be broadly categorized into two types: The first examines its economic effects. The residential environment is not only the physical space and residential conditions for individuals but also a vector for accessing resources like health and education services; the resulting economic effects are significant for individual welfare and regional development (5). In terms of environmental welfare, governance can promote improved living conditions, enhance resource efficiency, reduce pollutant emissions (6, 7), thus elevate environmental welfare performance (8). Regarding employment and income, such governance boosts human capital reserves, expands job opportunities, encourages non-agricultural employment, and raises resident income levels (9, 10). At the regional development level, enhanced residential environment increases the settlement willingness of migrant populations, accelerates the influx of high-skilled talent, and thereby stimulates regional economic growth (11). The second category explores the social effects of residential environment governance. On one hand, it can increase residents’ well-being by shortening commutes and promoting social participation, while also improving living quality and fostering a sense of belonging and mutual support (12, 13), thereby enhancing community governance efficiency through better residential infrastructure (14). On the other hand, governance based on natural environment restoration and urban environmental renovation can effectively improve residents’ health. The ecological environment is foundational for human survival; developing clean energy, optimizing energy consumption structures, and strengthening ecological civilization construction can all improve public health (15, 16). Urban environmental renovation can reduce obesity levels and the prevalence of chronic diseases such as cardiovascular ailments by improving the coverage of public infrastructure and increasing physical exercise (4, 17); they can also modify indoor residential environment to decrease fall risks for the older adults and maintain health (18). Fundamentally, the residential environment is a composite system of natural ecological and urban built environments. Isolating analysis to a single dimension makes it difficult to fully assess its actual value for residents’ health and may underestimate the interactive effects across different dimensions.

This study utilizes China’s “Ecological Restoration and Urban Renovation” (ERUR) pilot policy as a quasi-natural experiment to examine the effects of residential environment governance on residents’ health, its underlying channels, and its implications for residents’ medical expenditure. This study makes three main contributions: ① Methodologically, it leverages the exogenous policy shock of the pilot program to match pilot cities with micro-level survey data, employing a Difference-in-Differences (DID) model to estimate the micro-level health effects of residential environment governance. This approach mitigates estimation biases from reverse causality and omitted variables prevalent in prior research. ② Theoretically, it constructs a framework linking residential environment and resident health, systematically investigates the channels through which governance affects health, and extends the analysis to its impact on medical burden. ③ Practically, it conducts heterogeneity analysis on health benefits, exploring disparities across different regions and demographic groups, thereby providing an empirical basis for fostering a new development paradigm where environmental sustainability and public health are mutually reinforcing.

## 2 Policy background and research hypotheses

### 2.1 Policy background

In April 2015, the Central Committee of the Communist Party of China and the State Council issued the *Opinions on Accelerating Ecological Civilization Construction*, which highlighted that China’s level of ecological advancement still lags behind its socio-economic development. It emphasized ecological conservation and restoration shall mainly rely on natural recovery, supplemented by artificial intervention (19). In March 2017, the Ministry of Housing and Urban–Rural Development of the People’s Republic of China published the *Guiding Opinions on Strengthening Ecological Restoration and Urban Renovation Work*. This document reaffirmed that despite remarkable achievements in China’s urbanization, major challenges remain. These include severe environmental pollution, ecosystem degradation, inadequate infrastructure and a lack of public services. It identified the ERUR initiative as a key measure to improve the residential environment. This policy innovation focuses on enhancing environmental quality, addressing urban infrastructure deficiencies, and raising the standard of public services, representing a key practice for advancing environmental governance capabilities and transforming urban development patterns.

Sanya was designated as China’s first pilot city for the ERUR in June 2015. Subsequently, in March and July 2017, 19 cities (including Fuzhou) and 38 cities (including Baoding) were, respectively, listed as the second and third batches of pilot cities. “Ecological Restoration”(ER) targets air and water pollution control, restoration of abandoned land, and enhancement of the green space system to substantially improve the ecological environment. “Urban Renovation” (UR) focuses on expanding public spaces, improving transport conditions, upgrading public service capacity, and renovating old residential quarters to comprehensively enhance urban functionality. This policy practice has proven an effective measure for improving environmental quality and bridging infrastructure gaps, providing an ideal exogenous shock for investigating the micro-level health effects of residential environment governance.

### 2.2 Research hypothesis

The Grossman health demand model posits that individual health is determined by initial health capital, subsequent health investments, and the depreciation of health capital (20). The rate of health capital depreciation represents the speed of degradation of an individual’s health level, influenced by innate endowment, age, and external environmental factors, among others. The governance of the residential environment can be conceptualized as a form of health investment undertaken jointly by the government and individuals. This governance work is hypothesized to affect residents’ health status by reducing the depreciation rate of health capital. This linkage is plausible because the depreciation rate of health capital is significantly influenced by the residential environment (21). A more hygienic and accessible residential environment, fostered by effective residential environment governance, is likely to reduce residents’ disease prevalence, thereby lowering the health capital depreciation rate.

Residential environments comprise both natural settings (air quality, green resources) and social facilities (public spaces, infrastructure). It constitutes not only the physical space and housing conditions in which individuals reside but also serves as an important channel for accessing social resources like healthcare services and health knowledge. Therefore, it contributes greatly to residents’ health and well-being (5). Furthermore, environmental exposure theory suggests that various environmental pollutants within the residential environment constitute important sources of environmental exposure. Chronic or intermittent exposure to these pollutants through inhalation, dermal contact, and dietary intake can lead to elevated health risks. Mitigating the health risks associated with such exposure contributes to enhancing residents’ health and well-being (22). Social support theory also highlights that public spaces and infrastructure within the residential environment are key venues for residents to engage in social interactions and build social networks. Well-designed public spaces facilitate the formation of residents’ social support networks, while robust infrastructure not only improves access to health services but also reduces the cost of physical exercise and encourages higher exercise frequency (23, 24). Based on this theoretical framework, this study proposes Hypothesis 1:

*H1*: Governance of residential environment can effectively improve residents’ health levels.

Residential environment governance influences residents’ health and well-being through three channels. First, its foundational aspect lies in the natural ecological environment restoration. Air pollution not only elevates the prevalence of chronic conditions like respiratory diseases and cardiovascular ailments but also accelerates health capital depreciation and increases mortality rates across all age groups (25, 26). Conversely, green space resources provide essential venues for physical activity and social interaction while also effectively mitigating air pollution levels, thereby improving public health (27). Governance aimed at residential environment can enhance vegetation coverage for air purification through ecological restoration, optimize urban industrial layouts with pollution control to reduce emissions, and improve regional green space systems to expand coverage, culminating in a more favorable natural environment.

Second, residential environment governance centers on urban built environment renovation. The expansion and upgrading of public spaces, such as the establishment of green parks and sports facilities, enriches recreational opportunities, lowers the barriers to physical exercise, expands social support networks, and encourages proactive health behaviors (28). Simultaneously, enhancements in medical and sports infrastructure, like the development of primary healthcare centers and rural cultural plazas, shorten the distance to healthcare services, improve accessibility, reduces barriers to physical activity, and promote regular exercise. This collectively contributes to the creation of high-quality living spaces (29).

Third, residential environment governance aids in improving public health literacy. The cultivation of preventative health awareness relies on both accessible healthy environments and the behavioral modeling effect of peers within a community. On one hand, this type of governance helps upgrade health service facilities. It brings professional services such as health consultation and monitoring into daily life. Residents can therefore easily access professional guidance and broaden their health knowledge (30). On the other hand, residential environment governance helps build diverse health communication scenarios. Health education campaigns at public spaces and primary care facilities subtly equip residents with knowledge on chronic disease prevention and lifestyle management. This drives the adoption of preventive behaviors and improves population health. This nurtures the adoption of proactive preventative behaviors, ultimately enhancing population health. Based on this theoretical framework, this study proposes Hypothesis 2:

*H2*: residential environment governance can improve public health by restoring the ecological environment, upgrading infrastructure and improving health awareness.

## 3 Materials and methods

### 3.1 Empirical model

To assess improvements in the residential environment at the city level, the ERUR in China was used as a proxy for residential environment improvement. To evaluate the causal impact of these residential environment improvements on residents’ health and well-being, a DID model was employed. Cities designated as the ERUR pilot cities were treated as the treatment group, with all other cities serving as the control group. The following DID model was constructed:

$$\text{Healt}h_{\mathrm{ict}}=\beta_{0}+\beta_{1}\text{trea}t_{\mathrm{ic}}\times \mathrm{pos}t_{\mathrm{it}}+\beta_{2}X_{\mathrm{ict}}+\eta_{t}+\delta_{c}+\varepsilon_{\mathrm{ict}}$$ (1)

In this Equation 1, *Health_ict_* denotes the health status of individual *i* in city *c* in year *t*. The variable *treat_ic_* indicates whether city *c*, where individual *i* resides, was designated as a trial site for the ERUR, taking the value 1 if so and 0 otherwise. The dummy variable *post_it_* signifies the policy implementation period, equal to 1 after the trial establishment and 0 before. *X_ict_* encompasses individual-level and city-level control variables. *η_t_* represents year fixed effects, *δ_c_* denotes city fixed effects, and *ε*_ict_ is the error term. The coefficient *β*_1_ on the interaction term captures the impact magnitude of the residential environment policy on residents’ health.

### 3.2 Data sources

The data consist of two components. Individual-level data were drawn from the China Health and Retirement Longitudinal Study (CHARLS) covering the 2011–2020 survey waves. Covering 28 provinces and 125 cities in China, this dataset is highly representative. It surveys individuals aged 45 and above, providing multi-dimensional health indicators such as chronic diseases, activities of daily living, cognitive function, and mental health, which offer robust support for our analysis. City-level data were sourced from the China City Statistical Yearbook. It is worth noting that the ERUR was implemented in three batches (June 2015, March 2017, and July 2017). The timing of the five waves of CHARLS data aligns well with the research requirements.^1^

In line with the study’s objectives and data characteristics, we performed the following sample preprocessing steps: First, individuals under the age of 45 were excluded to focus on the middle-aged and older adults. Second, based on the city information of CHARLS respondents, we matched individuals with cities designated as the ERUR pilots, resulting in a treatment group comprising 22 cities. Third, observations with missing key variables or extreme outliers were removed. The final dataset comprises a panel covering five waves (2011, 2013, 2015, 2018, and 2020), yielding 36,386 valid person-wave observations from 10,464 unique individuals (15,118 observations for males; 21,268 observations for females).

### 3.3 Variable definitions

#### 3.3.1 Dependent variable

This study’s dependent variable is an individual’s health status. Given that no universally accepted measure of health status exists, prior research has often relied on single-dimensional metrics such as self-reported health, activities of daily living, or indicators of psychological well-being. To comprehensively evaluate the micro-level health effects of residential environment governance, this study takes a multi-dimensional approach, constructing a Comprehensive Health Index (CHI) from three distinct dimensions: physical health, cognitive capacity, and mental health.(1) Physical Health: 11 questionnaire items were used: the ability to independently perform six basic Activities of Daily Living (ADLs: dressing, bathing, eating, getting in/out of bed, using the toilet, controlling urination/defecation) and five Instrumental Activities of Daily Living (IADLs: doing housework, preparing meals, shopping, taking medicines, managing finances). Responses were coded as 1 (“no difficulty”), 2 (“some difficulty but can complete”), 3 (“needs assistance”), or 4 (“unable to complete”). A total score was calculated and normalized. (2) Cognitive Capacity: 10 items from the questionnaire were used: five questions on temporal orientation (e.g., ability to correctly identify the current year, month, date, day of week, and season) and five consecutive serial subtractions of 7 from 100 (or other appropriate subtraction). An incorrect answer was scored as 1. The total score across the 10 items was then normalized. (3) Mental Health: 10 items adapted from the Center for Epidemiologic Studies Depression Scale (CES-D) were used. Responses of “rarely or none of the time,” “some or a little of the time,” “occasionally or a moderate amount of the time,” and “most or all of the time” were assigned values of 1, 2, 3, and 4, respectively. The scores for the two positively worded items (“I felt hopeful about the future” and “I was happy”) were reverse-coded. The total score across all 10 items was normalized. Finally, the scores of the three dimensions are summed to obtain the CHI, which ranges from 0 to 3. It is a reverse indicator, where a higher score indicates poorer health status. For robustness checks, alternative health indicators—specifically the presence of chronic disease(s), a separate cognitive capacity score, and a separate mental health score—are used to replace the CHI, thereby ensuring the reliability of the research findings.

#### 3.3.2 Independent variable

The explanatory variable is the policy interaction term *treat_ic_* × *post_it_*. It quantifies the divergence in the change of residents’ health statuses—before and after the pilot initiative—between the treatment group and the control group. Considering that the CHARLS project was launched in July of 2011, 2013, 2015, 2018, and 2020, and aligning with the timing of the ERUR, the post-treatment observation points are defined as March 2017 and July 2017.

#### 3.3.3 Control variables

Following established research methodologies (31), this study incorporates control variables at two distinct levels. At the individual level, we account for demographic characteristics (age, gender, educational, marital status, and household registration type), lifestyle and behavioral factors (sleep duration and smoking/alcohol habits), and social security coverage (medical insurance and pension insurance). At the city level, we control for regional development level, regional population size, and regional medical resources. Please refer to Table 1 for descriptive statistical results.

**Table 1:** Descriptive statistics for the main variables.

Variable	Variable definition	Total sample (*N =* 36,386)		Treatment group (*N* = 3,166)		Control group (*N* = 33,220)	
		Mean	SD	Mean	SD	Mean	SD
CHI	Comprehensive health index	1.200	0.362	1.074	0.366	1.212	0.359
DID	Yes = 1; No = 0	0.087	0.282	1.000	0.000	0.000	0.000
Age	Measured in 5-year intervals	2.701	1.747	3.245	1.660	2.649	1.746
Gender	Male = 1, Female = 0	0.416	0.493	0.408	0.491	0.416	0.493
Education	Primary or below = 0; Junior high = 1; High school or above = 2	0.405	0.665	0.540	0.727	0.392	0.657
Marital status	Married/cohabiting = 1; Others = 0	0.809	0.393	0.793	0.405	0.811	0.392
Household registration type	Rural = 1; Urban = 0	0.791	0.407	0.687	0.464	0.801	0.399
Sleep duration	Continuous variable (hours)	6.034	1.945	6.054	1.831	6.032	1.956
Smoke	Yes = 1, No = 0	0.242	0.428	0.223	0.416	0.244	0.430
Alcohol	Frequently = 1; Occasionally = 2; Never = 3	2.470	0.838	2.483	0.833	2.469	0.839
Pension insurance	Yes = 1, No = 0	0.638	0.481	0.893	0.310	0.614	0.487
Medical insurance	Yes = 1, No = 0	0.955	0.208	0.974	0.158	0.953	0.212
Regional development level	GDP per capita (log)	10.715	0.545	11.216	0.446	10.668	0.529
Regional population size	Registered population (log)	6.210	0.625	6.222	0.597	6.208	0.627
Regional medical resources	Number of licensed physicians (log)	9.276	0.742	9.765	0.743	9.229	0.724

## 4 Results

### 4.1 Benchmark regression results

Table 2 presents the benchmark regression results regarding how residential environment governance affects residents’ health. Column (1) presents estimates without controlling for fixed effects or covariates. Columns (2) through (4) control for year and city fixed effects while sequentially introducing individual and city level control variables. Since the CHI is a reverse indicator, all regression coefficients of the ERUR are significantly negative, indicating that policy implementation improves residents’ health level by an average of 1.80% in pilot cities relative to non-pilot cities. This demonstrates that residential environment governance generates a significant health-promoting effect. First, residential environment governance helps improve air quality and reduce pollution via air and water pollution control measures, which contributes to better residents’ health (8, 32). Furthermore, by increasing green space area and improving the green space system, it effectively moderates regional temperature and humidity, creating comfortable living spaces, thereby boosting health outcomes. Second, such governance improves infrastructure and living conditions. It also enlarges urban public spaces, encouraging residents to engage more in social activities and physical exercise. This helps foster healthy lifestyles and lift residents’ health level. The above findings offer preliminary evidence to support Hypothesis H1.

**Table 2:** Benchmark regression results.

Variable	(1) CHI	(2) CHI	(3) CHI	(4) CHI
DID	−0.138^***^ (0.009)	−0.016^**^ (0.008)	−0.021^***^ (0.007)	−0.018^**^ (0.008)
Age			0.024^***^ (0.002)	0.024^***^ (0.002)
Gender			−0.128^***^ (0.006)	−0.128^***^ (0.006)
Education			−0.109^***^ (0.004)	−0.109^***^ (0.004)
Marital status			−0.046^***^ (0.006)	−0.046^***^ (0.006)
Household registration type			0.110^***^ (0.007)	0.110^***^ (0.007)
Sleep Duration			−0.025^***^ (0.001)	−0.025^***^ (0.001)
Smoke			0.020^***^ (0.006)	0.020^***^ (0.006)
Alcohol			0.013^***^ (0.003)	0.013^***^ (0.003)
Pension insurance			−0.029^***^ (0.005)	−0.029^***^ (0.005)
Medical insurance			−0.091^***^ (0.009)	−0.091^***^ (0.009)
Regional development level				0.023^**^ (0.011)
Regional population size				0.064^**^ (0.031)
Regional medical resources				−0.036^***^ (0.010)
_cons	1.212^***^ (0.003)	1.202^***^ (0.003)	1.401^***^ (0.017)	1.092^***^ (0.219)
*N*	36,386	36,386	36,386	36,386
*Adj-R* ^2^	0.016	0.127	0.281	0.281
Year FE	No	Yes	Yes	Yes
City FE	No	Yes	Yes	Yes

** and *** denote significance at 5% and 1% levels. Robust standard errors are in parentheses.

The results for the control variables are largely consistent with the theoretical understandings established in existing health research. Regarding demographic characteristics, resident health gradually worsens as age increases. Gender, household registration status and marital status exert significant effects. Health status is better among males and urban residents than among females and rural residents, and married individuals have superior health. Education demonstrates a significant protective effect on individual health (33). In terms of personal behavioral traits, longer sleep duration boosts immunity and improves health, while smoking and alcohol consumption damage body organs and are detrimental to resident health. Concerning social security factors, pension insurance and medical insurance improve health by enhancing economic stability and healthcare accessibility. For socioeconomic characteristics, growth in regional population size can strain healthcare resources and lower service accessibility, which hinders improvements in residents’ health. Conversely, regions with abundant medical resources can deliver high-quality and convenient healthcare services, which helps improve residents’ health.

### 4.2 Endogeneity tests

#### 4.2.1 Parallel trend test

The validity of benchmark results relies on pre-trend test evidence. Before the policy shock, the treatment and control groups showed similar trends in residents’ health. Drawing on prior research, the event study method was employed to test whether the parallel trends were satisfied, with the regression equation presented in Equation 2:

$$\text{Healt}h_{\mathrm{ict}}=\alpha +\beta_{t}\sum_{t=-2}^{2}\text{trea}t_{\mathrm{ic}}\times \mathrm{pos}t_{\mathrm{it}}+\gamma X_{\mathrm{ict}}+\eta_{t}+\delta_{c}+\varepsilon_{\mathrm{ict}}$$ (2)

**Figure 1:**
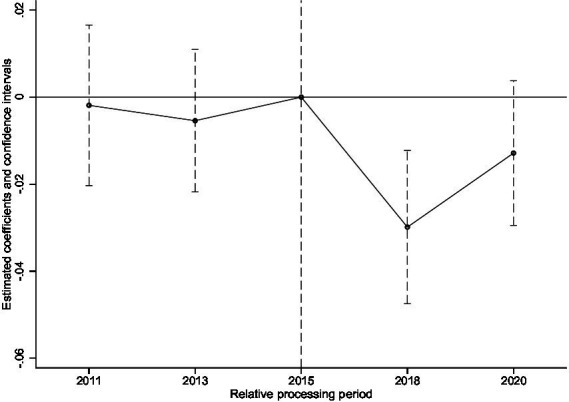
Parallel trend test.

Here, *treat_ic_ × post_it_* represents a dummy variable for the year t before and after the policy implementation, with *β*_t_ indicating the difference in residents’ health levels between the treatment and control groups t years after the policy was implemented. We take 2015, the latest pre-policy observation year, as the baseline for the parallel trend test. The ERUR was launched in 2017, and this study adopts five waves of unbalanced panel data (2011, 2013, 2015, 2018, 2020). Taking 2015 as the reference enables an objective comparison of health trends before and after policy implementation. Figure 1 presents the estimation results at the 90% confidence interval. Before the ERUR took effect, the regression coefficients of 2011 and 2013 fluctuate around zero. This suggests the CHI shared consistent trends across regions prior to policy implementation, which validates the parallel trend assumption. Remarkable policy effects emerged in 2018 yet weakened in 2020. On the one hand, local governments boosted investment in residential environment improvement to meet assessment targets in the early pilot stage, yielding prominent short-term health benefits. As investment gradually slowed down, the marginal policy effect declined accordingly. On other hand, the launch of Healthy China 2030 Initiative promoted nationwide health development. The health status of residents in non-pilot areas improved synchronously, which reduced the policy impact in 2020.

#### 4.2.2 Random sampling placebo test

**Figure 2:**
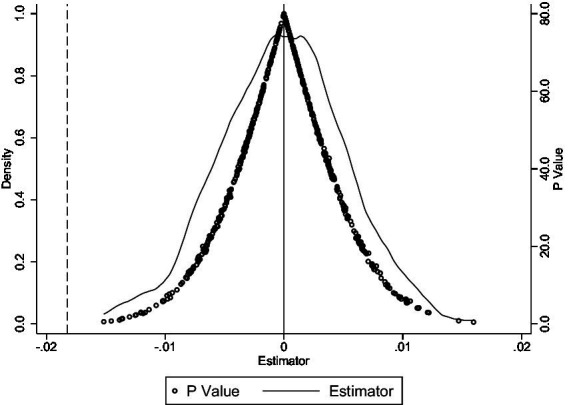
Sample random reconstruction placebo test.

Given that our pilot city sample represents a relatively small proportion (approximately 8.70%) of the total sample, careful consideration must be given to whether other incidental factors might account for the observed differences in residents’ health between the treatment and control groups after 2017. To address this, we adopted the methodology used by Topalova (34). We performed random sampling without replacement to create a pseudo-treatment group consisting of the same number of individuals. This randomization procedure was repeated 500 times to conduct regression analyses. The distribution of the estimated coefficients from these analyses is plotted in Figure 2. The figure shows that the pseudo-regression coefficients follow a normal distribution with a mean of zero, which is significantly different from the coefficient of independent variable (−0.018) in our baseline regression. This finding demonstrates that the impact of residential environment governance on residents’ health levels is not attributable to random chance, lending high credibility to the baseline regression results.

#### 4.2.3 Random timing placebo test

To ensure health differences between the treatment and control groups were not driven by temporal trends, and considering CHARLS’s biennial survey design, we moved the ERUR implementation forward by 3 and 5 years. Specifically, we assumed the policy was implemented in 2014 and 2012, and performed regressions with these fictitious policy timelines. As shown in columns (1) and (2) of Table 3, the estimated coefficients for the policy pilot failed to reach statistical significance at the 10% level in both cases. This indicates that, absent the actual policy shock from the ERUR, no systematic differences in resident health outcomes would have been observed.

**Table 3:** Time placebo test and PSM-DID regression results.

Variable	(1) CHI	(2) CHI	(3) CHI
DID*_2014_*	−0.010 (0.008)		
DID*_2012_*		−0.010 (0.010)	
DID*_psm_*			−0.015^*^ (0.008)
_cons	1.092^***^ (0.219)	1.095^***^ (0.220)	1.277^***^ (0.259)
*N*	36,386	36,386	28,405
*Adj-R* ^2^	0.281	0.281	0.269
Control	Yes	Yes	Yes
Year FE	Yes	Yes	Yes
City FE	Yes	Yes	Yes

* and *** denote significance at 10 % and 1% levels. Robust standard errors are in parentheses.

#### 4.2.4 PSM-DID robustness test

The phased rollout of the pilot program may have introduced selection bias due to the anticipation of policy benefits, potentially leading to inherent health disparities between residents in pilot and non-pilot cities. To address this, a PSM-DID approach was employed. Using resident health as the outcome variable and the original control variables as covariates, a 1:1 nearest neighbor matching method was applied to re-select the analysis sample. The regression results, presented in column (3) of Table 3, show that the estimated policy coefficient remains statistically significant at the 10% level. This confirms that the health promoting effect of residential environment governance persists significantly even after matching.

### 4.3 Robustness tests

Both the benchmark regressions and endogeneity tests indicate that implementing residential environment governance can enhance residents’ health. To rule out interference from other factors affecting the conclusion, robustness tests are required to improve the reliability of the findings.

#### 4.3.1 Replacing the dependent variable

To ensure the robustness of estimation results, this study separately replaces the CHI with the prevalence of chronic diseases, cognitive ability, and depressive status before re-conducting empirical tests. The results are shown in columns (1) through (3) of Table 4. As shown, the ERUR continues to reduce the prevalence of chronic diseases such as hypertension and diabetes among residents, as well as improve individual cognitive ability and mental health. All results pass significance tests at a minimum statistical level of 5%, indicating that the positive effect of residential environment governance on residents’ health is relatively robust.

**Table 4:** Robustness test I.

Variable	(1) Chronic diseases	(2) Cognitive ability	(3) Depressive status
DID	−0.027^**^ (0.012)	−0.142^**^ (0.056)	−0.297^**^ (0.141)
_cons	0.055 (0.319)	3.787^**^ (1.657)	17.296^***^ (4.024)
*N*	36,386	36,386	36,386
*Adj-R* ^2^	0.105	0.256	0.168
Control	Yes	Yes	Yes
Year FE	Yes	Yes	Yes
City FE	Yes	Yes	Yes

** and *** denote significance at 5% and 1% levels. Robust standard errors are in parentheses.

#### 4.3.2 Incorporating additional fixed effects

To effectively control the impact of time-invariant omitted variables at different levels on estimation results, this study adds individual, household, and community fixed effects to the baseline model, as shown in columns (1) to (3) of Table 5. Furthermore, to eliminate potential interference from time-varying omitted variables, province-year interaction fixed effects are incorporated into the baseline model, effectively controlling for most regional and time-varying potential confounding factors. The regression results are presented in column (4) of Table 5. The results show that potential omitted variables do not substantially affect the impact of the ERUR on residents’ health, and the baseline regression results remain robust.

**Table 5:** Robustness test II.

Variable	(1) CHI	(2) CHI	(3) CHI	(4) CHI	(5) CHI	(6) CHI
DID	−0.019^***^ (0.007)	−0.020^***^ (0.007)	−0.019^***^ (0.007)	−0.014^*^ (0.008)	−0.018^**^ (0.009)	−0.018^*^ (0.011)
_cons	0.844^***^ (0.209)	0.947^***^ (0.208)	1.109^***^ (0.218)	1.087^***^ (0.351)	1.092^***^ (0.292)	1.092^***^ (0.263)
*N*	36,386	36,386	36,386	36,386	36,386	36,386
*Adj-R* ^2^	0.615	0.530	0.298	0.283	0.281	0.281
Control	Yes	Yes	Yes	Yes	Yes	Yes
Year FE	Yes	Yes	Yes	Yes	Yes	Yes
City FE	Yes	No	No	Yes	Yes	Yes
Individuality FE	Yes	No	No	No	No	No
Family FE	No	Yes	No	No	No	No
Community FE	No	No	Yes	No	No	No
Province × Year FE	No	No	No	Yes	No	No

*, **, and *** denote significance at 10, 5, and 1% levels. Robust standard errors are in parentheses.

Columns (5) and (6) calculate robust standard errors at the community level and city level, respectively.

#### 4.3.3 Altering the robust standard error calculation method

The benchmark regressions compute robust standard errors clustered at the household level. To exclude the influence of all time-varying factors at the community and city levels, such as community policy measures and regional economic development. Drawing on Harari’s (35) research methodology, robust standard errors were calculated using cluster analysis at both community and city levels. The regression results are presented in columns (5) and (6) of Table 5. The results indicate that residential environment governance significantly improves residents’ health.

#### 4.3.4 Controlling for other policy interference

Existing research has found that policies such as the “China’s Healthy City” (CHC), the “Long-Term Care Insurance Pilot” (LTCI), and the “Ecological Civilization Pilot” (ECP) can effectively improve residents’ health (36–38). To isolate the influence of other policies on residents’ health during the sample period, this study introduces dummy variables for the CHC, the LTCI, and the ECP to control for the effects of these policies. The regression results, presented in columns (1) through (3) of Table 6, demonstrate that after accounting for the potential influence of these policies, the positive effect of residential environment governance on residents’ health remains robust.

**Table 6:** Robustness test III.

Variable	(1) CHI	(2) CHI	(3) CHI	(4) CHI	(5) CHI
DID	−0.0184^**^ (0.0076)	−0.0182^**^ (0.0075)	−0.0183^**^ (0.0075)	−0.0266^***^ (0.0096)	−0.0173^**^ (0.0075)
CHC	Control				
LTCI		Control			
ECP			Control		
_cons	1.0951^***^ (0.2237)	1.0661^***^ (0.2200)	1.0669^***^ (0.2213)	0.9210^***^ (0.2959)	1.0894^***^ (0.2187)
*N*	36,386	36,386	36,386	27,493	35,453
*Adj-R* ^2^	0.2813	0.2813	0.2813	0.2724	0.2798
Control	Yes	Yes	Yes	Yes	Yes
Year FE	Yes	Yes	Yes	Yes	Yes
City FE	Yes	Yes	Yes	Yes	Yes

** and *** denote significance at 5% and 1% levels. Robust standard errors are in parentheses.

#### 4.3.5 Alternative sample restrictions

First, considering the interference of public health emergencies with residents’ health levels and awareness of disease prevention, this study selects only the four waves of survey data from CHARLS 2011–2018 to exclude the potential impact of the COVID-19 pandemic on individual health, as shown in column (4) of Table 6. Second, municipalities directly under the central government possess higher administrative status, developed economies, and abundant medical resources, which may potentially influence the implementation of pilot policies. Therefore, a regression was rerun after removing samples from municipalities, as reported in column (5) of Table 6. The results show that residential environment governance significantly improves residents’ health status, whether public health emergencies or municipal samples are excluded.

### 4.4 Heterogeneity analysis

Considering the reality of differing individual characteristics and uneven distribution of regional medical resources, this section further analyzes the heterogeneous characteristics of how residential environment governance influences residents’ health.

#### 4.4.1 Urban–rural heterogeneity

Given the significant disparities in ecological conditions, infrastructure, and public services between urban and rural areas in China, this study investigates the urban–rural heterogeneity in the impact of residential environment governance on resident health. The regression results are presented in columns (1) and (2) of Table 7. The findings indicate that the ERUR has a significant effect only on urban residents’ health. While a positive effect is observed for rural residents, it is not statistically significant. This discrepancy comes from two aspects. First, the pilot policy of the ERUR targets central urban areas, old districts and aged built-up zones. The ERUR improves urban residents’ daily living, commuting and leisure conditions. It cuts urban environmental health risks and produces significant health benefits for urban residents. Second, urban and rural areas have fundamentally different demands for residential environment governance. Key rural governance tasks include toilet renovation, domestic waste disposal, domestic sewage treatment and rural landscape upgrading. These items are not included in the ERUR scope. Hence the ERUR hardly reduces health risks for rural residents. Only suburban villages near cities gain partial spillover benefits from urban residential environment governance and achieve slight health improvements. Nevertheless, the spillover has limited coverage and weak magnitude and shows no statistically significant results.

**Table 7:** Heterogeneity analysis I.

Variable	(1) City	(2) Rural	(3) Low-education group	(4) High-education group
	CHI	CHI	CHI	CHI
DID	−0.029^**^ (0.013)	−0.009 (0.009)	−0.019^**^ (0.008)	0.009 (0.019)
_cons	1.778^***^ (0.431)	0.990^***^ (0.262)	1.174^***^ (0.239)	0.134 (0.537)
*N*	7,608	28,778	32,727	3,659
*Adj-R* ^2^	0.221	0.250	0.252	0.180
Control	Yes	Yes	Yes	Yes
Year FE	Yes	Yes	Yes	Yes
City FE	Yes	Yes	Yes	Yes

** and *** denote significance at 5% and 1% levels. Robust standard errors are in parentheses.

Furthermore, China launched the Three-Year Action Plan for Improving Rural Human Settlements within our sample period. To mitigate potential confounding bias stemming from this nationwide policy, we add an extra control for rural settlement renovation effects in subsample regressions restricted to rural households.^2^ The regression results show that rural residential environment governance significantly improves rural residents’ health, whereas the health benefits of the ERUR policy remain statistically insignificant for rural groups. This evidence further confirms that the ERUR yields statistically significant health improvements solely among urban residents.

#### 4.4.2 Educational heterogeneity

The educational attainment of residents directly influences their health literacy, behavioral choices, and capacity to perceive the benefits of policies. This study further examines the educational heterogeneity in how residential environment governance affects resident health. Residents with junior high school education or below are defined as the low-education group, and those with high school education or above as the high-education group. Regression results are presented in columns (3) and (4) of Table 7. The analysis reveals that the ERUR exerts a statistically significant effect solely on residents’ health with lower educational attainment. This outcome can be attributed to the following: Residents with lower education levels tend to live in areas with poor environments and work low-skill jobs. Their poor working and living conditions bring higher health risks. In contrast, more educated residents have better housing and access to health resources, so the policy produces limited health improvements for them. Therefore, residential environment governance demonstrates a more pronounced health effect specifically for residents with lower levels of education.

#### 4.4.3 Heterogeneity in environmental livability

The environmental foundation exerts a significant influence on governance outcomes. Areas with a favorable environmental base, leveraging their resource endowments and developmental advantages, provide crucial support for the health effects of governance initiatives. This section further analyzes heterogeneity in the health impacts of residential environment governance based on environmental livability. Taking built-up area green coverage rate as the grouping criterion for regression analysis, regions with a green coverage rate below the median (40.38%) are classified as having lower livability, while others are classified as having higher livability. The regression results are presented in columns (1) and (2) of Table 8. The analysis reveals that the ERUR exhibits a significant health improvement effect only for residents in areas with higher environmental livability. This is primarily attributed to the fact that regions with higher livability possess a superior environmental base, higher health literacy, and more complete public infrastructure. These conditions enable residential environment governance to fully realize its health improvement effects through complementary mechanisms. Conversely, areas with lower livability are constrained by deficiencies in the foundational environment and fragmented policy resources, which hinder the residential environment governance from generating significant health benefits.

**Table 8:** Heterogeneity analysis II.

Variable	(1) High livability	(2) Low livability	(3) Abundant medical resources	(4) Lack medical resources
	CHI	CHI	CHI	CHI
DID	−0.020^*^ (0.011)	−0.015 (0.015)	0.001 (0.018)	−0.016^*^ (0.009)
_cons	1.572^***^ (0.467)	0.976^***^ (0.262)	−0.755 (0.967)	1.084^***^ (0.234)
*N*	18,243	18,143	3,428	32,958
*Adj-R* ^2^	0.255	0.287	0.223	0.279
Control	Yes	Yes	Yes	Yes
Year FE	Yes	Yes	Yes	Yes
City FE	Yes	Yes	Yes	Yes

* and *** denote significance at 10% and 1% levels. Robust standard errors are in parentheses.

#### 4.4.4 Heterogeneity in medical resources

The density of medical resource distribution and the accessibility of medical services are critical determinants of resident health. Improving infrastructure conditions and community environments can further amplify the health utility of medical resources. Therefore, this study further investigates the heterogeneity in the health impacts of residential environment governance based on medical resource availability. Provincial capital cities in China are defined as areas with abundant medical resources, while other regions are categorized as areas with relatively scarce medical resources. The regression results are shown in columns (3) and (4) of Table 8. The findings indicate that the ERUR has a significant promoting effect on resident health in areas with scarce medical resources. Regions abundant in medical resources are predominantly concentrated in urban cores where the residential environment is generally satisfactory, and residents’ health awareness and literacy are relatively high. Consequently, governance interventions struggle to surpass the existing ceiling of health outcomes. In contrast, areas scarce in medical resources suffer from a weak environmental foundation, allowing investments in residential environment governance to be effectively translated into measurable health gains, thereby producing a pronounced health promotion effect.

### 4.5 Analysis of transmission channel

Based on previous theoretical analysis and benchmark regression results, this study adopts the method of David (39), It explores the transmission channels between residential environment governance and residents’ health from ecological environment restoration, infrastructure upgrading and health awareness improvement.

The first is the ecological environment restoration channel. Drawing on the research of Hanna and Oliva and Lelieveld et al. (40, 41), PM2.5 and PM10 annual average concentrations were selected to measure air pollution levels, while urban green space area and park green space area were used to assess ecological restoration. These metrics were incorporated into the baseline model as mediating variables. The regression results are presented in Table 9. Columns (1) and (2) show that the policy coefficients are significantly negative at the 1% statistical level, indicating that residential environment governance effectively reduces regional PM2.5 and PM10 concentrations. In columns (3) and (4), the policy coefficients are significantly positive at the 1% level, demonstrating that such governance contributes to increasing regional green space and park green space area. Thus, residential environment governance can reduce air pollution, restore the ecological environment, and consequently improve residents’ health levels. The underlying rationale is that this governance work both controls pollutant emissions at the source and restores degraded ecological spaces by expanding urban green areas. Leveraging the synergistic purification effects of air and greenery, it provides residents with a high-quality environment, thereby enhancing public health (27).

**Table 9:** Ecological environment restoration channels.

Variable	(1) PM2.5	(2) PM10	(3) Green area	(4) Park green space area
DID	−1.007^***^ (0.3044)	−1.534^***^ (0.3778)	3.844^***^ (1.1659)	2.016^***^ (0.5414)
_cons	102.299^***^ (8.1845)	61.298^***^ (8.3984)	−795.292^***^ (49.6615)	−392.924^***^ (23.2873)
*N*	36,386	36,386	36,386	36,386
*Adj-R* ^2^	0.887	0.958	0.977	0.932
Control	Yes	Yes	Yes	Yes
Year FE	Yes	Yes	Yes	Yes
City FE	Yes	Yes	Yes	Yes

*** denote significance at 1% level. Robust standard errors are reported in parentheses.

The second is the infrastructure upgrading channel. This study selects personal residential renovation and public activity space renovation as channel variables for analysis. Based on CHARLS survey data, residential renovation is measured using whether homes have ‘installed bathing facilities,’ ‘installed flush toilets,’ and ‘accessed broadband internet’; a value of 1 is assigned if all conditions are met, otherwise 0. Due to the absence of direct observation of activity space renovation in CHARLS, it is indirectly measured through changes in participation in physical exercise and social activities, using ‘engagement in moderate to low-intensity physical activities’ and ‘playing mahjong, cards, chess, or visiting community activity centers’; a value of 1 is assigned if these activities occur, otherwise 0. The regression results are shown in columns (1)–(2) of Table 10. The regression coefficient in column (1) is 0.035, significant at the 1% level. This shows that residential environment governance, including renovating old communities and upgrading housing, facilitates residential renewal and brings convenience to residents. The regression coefficient in column (2) is 0.017, meaning residential environment governance can significantly promote the renovation of public activity spaces. This is attributed to its facilitation of utilizing idle land and renovating old plazas, accelerating the construction of community renovation projects and sports parks, thereby creating more expansive spaces for residents’ physical exercise and social interaction. It is evident that residential environment governance can improve individual health levels via personal residential and public activity space renovation.

**Table 10:** Infrastructure upgrading channels and health awareness improvement channels.

Variable	(1) Personal residential renovation	(2) Public activity space renovation	(3) Chronic disease screen	(4) Promotion of chronic disease knowledge	(5) Chronic disease treatment
DID	0.035^***^ (0.011)	0.017^**^ (0.007)	0.010^**^ (0.005)	0.011^**^ (0.005)	0.007^**^ (0.004)
_cons	0.488^**^ (0.246)	0.099 (0.192)	0.149 (0.147)	0.123 (0.154)	0.517^***^ (0.111)
*N*	36,386	36,386	48,824	48,824	48,824
*Adj-R* ^2^	0.229	0.028	0.029	0.030	0.026
Control	Yes	Yes	Yes	Yes	Yes
Year FE	Yes	Yes	Yes	Yes	Yes
City FE	Yes	Yes	Yes	Yes	Yes

* and *** denote significance at 5% and 1% levels. Robust standard errors are in parentheses.

The third is the health awareness improvement channel. China, having the world’s largest diabetic population—approximately one-quarter of global cases—is primarily affected by genetic factors, poor dietary habits, obesity, and lack of exercise (42). Utilizing CHARLS survey data, chronic disease screening is measured by ‘use of urine glucose test, fundus examination, or microalbuminuria test for blood glucose monitoring’; chronic disease knowledge dissemination by ‘whether a doctor provided health education on weight control and physical exercise for diabetic patients’; and chronic disease treatment by ‘use of traditional Chinese medicine or insulin for diabetes management.’ A value of 1 is assigned if the activity occurred, otherwise 0. The regression results are shown in columns (3)–(5) of Table 10. All policy coefficients are significantly positive, signifying that residential environment governance increases the frequency of chronic disease screening, enhances the dissemination of chronic disease knowledge, and promotes chronic disease treatment, thereby effectively boosting residents’ disease prevention awareness. On one hand, the ERUR renovates urban public spaces and integrates health education initiatives. For example, parks are fitted with signs about daily exercise and chronic disease prevention, and community health centers provide consultations and wellness services. This makes health knowledge easier for residents to access. On the other hand, leveraging community venues and rural cultural plazas, the policy facilitates the dissemination of health and prevention ideas during residents’ social and wellness activities. It encourages more residents to monitor their health and strengthen disease prevention awareness. Consequently, research hypothesis H2 is validated.

## 5 Further expansion: impact of residential environment governance on healthcare burden

Resident health is closely linked to healthcare burden. The aforementioned research suggests that improving residential environment can significantly enhance residents’ health; consequently, its potential impact on healthcare affordability merits investigation. To comprehensively assess the influence of residential environment governance on public well-being, this analysis proceeds by examining healthcare utilization and medical expenditure.

Theoretically, residential environment governance could reduce health risks at their source, decrease demand for medical services, and reduce healthcare utilization (43, 44). This study employs three metrics to measure the use of health services: whether the respondent visited an outpatient facility or received home care services in the past month; whether they were hospitalized in the past year; and whether they self-medicated in the past month. Utilization of outpatient, inpatient, or self-treatment services is coded as 1; otherwise, it is 0. The empirical results, presented in Table 11, indicate that the policy pilot significantly increases healthcare service utilization. Specifically, after controlling for either individual fixed effects or city fixed effects, residents in pilot cities show a 2.30% higher probability of using medical services compared to those in non-pilot cities. Therefore, contrary to prior findings, the governance intervention did not reduce residents’ demand for treatment or their utilization of healthcare services.

**Table 11:** Impact effect of residential environment governance on medical service utilization.

Variable	(1) Acceptance of medical services	(2) Acceptance of medical services
DID	0.023^*^ (0.013)	0.027^**^ (0.013)
_cons	0.584 (0.411)	0.500 (0.412)
*N*	27,380	27,380
*Adj-R* ^2^	0.049	0.213
Control	Yes	Yes
Year FE	Yes	Yes
City FE	Yes	No
Individual FE	No	Yes

* and *** denote significance at 10% and 5% levels. Robust standard errors are in parentheses.

Furthermore, this study attempts to analyze the impact of residential environment governance on residents’ outpatient expenditure, hospitalization expenditure, and self-medication expenditure. Outpatient expenditure are measured using the item: “Approximately what was your total outpatient medical expense in the past month? (including both out-of-pocket and reimbursed portions).” Hospitalization expenditure are measured with: “Approximately what was your total hospitalization expense in the past year? (including both out-of-pocket and reimbursed portions).” Self-medication expenditure are measured with: “Approximately how much did you spend on self-purchased medication in the past month? (including both out-of-pocket and reimbursed portions).” The regression results are presented in Table 12. The findings indicate that residential environment governance did not have a statistically significant impact on residents’ outpatient or hospitalization expenditure. However, it exerted a significant positive effect on self-medication expenditure. Specifically, residents in the pilot cities experienced a 13.60% increase in self-medication expenditure compared to residents in non-pilot cities. Extended analysis reveals that residential environment governance did not directly reduce residents’ utilization of medical services or lower their healthcare financial burden. Conversely, the ERUR increased residents’ self-medication expenditure.

**Table 12:** Impact effect of residential environment governance on medical expenditure.

Variable	(1) Outpatient expenditure	(2) Hospitalization expenditure	(3) Self-medication expenditure
DID	0.003 (0.059)	0.088 (0.078)	0.136^*^ (0.079)
_cons	5.606^***^ (1.952)	1.632 (2.054)	0.871 (2.239)
*N*	27,380	27,380	27,380
*Adj-R* ^2^	0.026	0.025	0.091
Control	Yes	Yes	Yes
Year FE	Yes	Yes	Yes
City FE	Yes	Yes	Yes

* and *** denote significance at 10% and 1% levels. Robust standard errors are in parentheses.

A plausible explanation is twofold. On one hand, the rising cost does not stem from deteriorated health conditions. Improved primary medical facilities make medical care more accessible, which increases residents’ medical visits and self-medication purchases. More local medical centers are built along with environmental improvement, lowering access barriers and pushing up self-diagnosis expenses. On the other hand, better residential conditions raise public health awareness. Residents shift from passive treatment to active prevention. Growing proactive health demands further lift relevant spending. No obvious changes are observed in outpatient and hospitalization expenses. It further proves that the policy exerts little impact on healthcare utilization for severe illnesses. The spending growth mainly reflects enhanced awareness of disease prevention and health management.

## 6 Conclusion and recommendations

Based on the intrinsic relationship between residential environment governance and residents’ health, this study constructs a theoretical analysis framework. Using five waves of CHARLS survey data from 2011 to 2020 and the ERUR pilot policy, it systematically identifies how residential environment governance affects residents’ health and the underlying channels. Our research yields several key findings. First, residential environment governance significantly improves residents’ health levels—a core conclusion that remains robust following endogeneity discussions and a series of robustness checks. Second, this governance enhances public health through three primary channels: ecological environment restoration, infrastructure upgrades, and health awareness improvement. Third, the health effects of such governance demonstrate significant heterogeneity. They not only greatly improve the health of urban residents and low-education groups, but also exert stronger effects in areas with high livability and limited medical resources. Finally, extended analyses reveal that residential environment governance does not directly reduce the utilization of medical services or lower residents’ medical burden. Paradoxically, it leads to an increase in residents’ expenditures for self-diagnosis and healthcare. The essence of this phenomenon is not an irrational expansion of total medical demand. Instead, it reflects rising health awareness and upgraded healthcare demand structure among residents.

These findings hold significant implications for advancing residential environment governance and elevating public health standards.

First, accelerate the improvement of residential environment to enhance living conditions. Environmental governance plays a significant role in improving residents’ health outcomes. Therefore, it is essential to transform pilot experiences in the ERUR into routine policies, continuously refine implementation guidelines and evaluation systems for pilot programs, and consider incorporating health indicators into environmental policy assessment criteria. Concurrently, establish cross-departmental coordination channels to integrate resources from ecological conservation, urban development, and public health authorities, ensuring effective policy implementation and elevating the quality of residential environment improvements.

Second, close coordination among multi-level departments underpins health promotion. As revealed by the channel analysis, such governance demands joint efforts across administrative levels. Regionally, this entails increased investment in comprehensive pollution control and ecological restoration to improve environmental quality. At the community level, leveraging policies for old residential area renovation can accelerate upgrades to personal dwellings and public spaces, optimizing residential environment. Individually, health education campaigns focused on disease prevention, conducted through community health centers, are vital for strengthening proactive health awareness.

Third, address diverse health demands, balancing support for vulnerable groups and enhancing existing advantages. Heterogeneity analysis suggests policy optimization must address “shortcomings” while “leveraging strengths.” For disadvantaged areas such as rural regions and those with insufficient medical resources, governance efforts should be coupled with increased provision of health services, exploring context-specific policy models. In areas with higher baseline livability and education levels, the focus should shift towards upgrading health service functionalities and deepening health education to amplify the positive health effects of environmental governance.

Fourth, improve the accessibility and affordability of primary medical services. Based on extended analysis, we pay attention to variations in medical service utilization and residents’ medical expenditure resulting from residential environment governance. On one hand, steadily build more grassroots medical stations to reduce residents’ travel costs for medical care, elevate service accessibility and satisfy needs for health management and daily consultation. On the other hand, refine the medical security system and broaden medical insurance coverage to ease residents’ medical expenses and health management costs.

This study systematically investigates the causal effect of residential environment governance on residents’ health and well-being. However, constrained by data availability and research experience, several limitations remain. First, the benchmark regressions construct a composite health indicator based on activities of daily living, cognitive function, and mental health, while an extended analysis examines how residential environment governance affects medical burdens. Nevertheless, residents’ health and well-being represent a multifaceted concept encompassing objective health metrics such as chronic diseases and physical function, subjective well-being dimensions including self-rated health and life satisfaction, as well as extended indicators related to healthcare utilization, labor supply, and social participation. Future research could develop a more comprehensive integrated indicator system to improve measurement accuracy and comprehensiveness. Second, the CHARLS survey design restricts our sample. All respondents are aged 45 or older. Thus we cannot analyze residents across the full life cycle. In terms of environmental health exposure, children and teenagers are at a critical growth stage. Residential environment strongly affects their physical and mental health. Working-age adults rely heavily on urban residential environment and public infrastructure for daily commuting and social activities. Residential environment governance also brings obvious health benefits to this group. Since CHARLS only covers middle-aged and older adults above 45, our results may underestimate the overall health gains of residential environment governance. Future research can add data from other micro surveys. It can accurately estimate policy effects on all age groups and explore heterogeneous impacts across different birth cohorts and age groups. In addition, CHARLS lacks survey data of Sanya, Hainan. This city, an initial pilot of the ERUR, is excluded from the research, which may lead to deviations in policy evaluation. More micro survey data will be adopted to optimize relevant analysis in follow-up studies. Third, this study utilizes the ERUR as an exogenous shock for identification. However, this policy’s environmental governance initiatives are more systematic and comprehensive at the city level, while its coverage and implementation intensity regarding rural residential environment improvements remain relatively limited. Although this study conducts heterogeneity analysis between urban and rural areas, it still struggles to fully distinguish the differential health effects of urban versus rural residential environment governance. Future research could employ more targeted policy shocks to separately examine the health impacts of urban renewal and rural residential environment improvement programs. Finally, the observation period for this study covers five datasets spanning from 2011 to 2020, with only two waves of post-intervention follow-up data, making it difficult to capture long-term cumulative effects and dynamic characteristics of governance. Both environmental improvements and the enhancement of health and well-being exhibit long-term and lag effects. Subsequent research could incorporate longer-term panel data to systematically reveal long-term effect.

## Data Availability

The datasets presented in this study can be found in online repositories. The names of the repository/repositories and number(s) can be found at: https://charls.charlsdata.com/pages/data/111/zh-cn.html.

## Footnotes

1 Since the first batch pilot of the ERUR was launched in Sanya in 2015, and CHARLS does not conduct surveys in Sanya, this paper only evaluates the health effects of the second and third batches of pilot cities.

2 Given the universal nationwide implementation of the policy without staggered regional rollouts, we cannot construct a standard policy dummy variable for regression identification. Since rural toilet upgrading stands as the core task of the initiative, we create a proxy variable leveraging CHARLS survey information based on household toilet conditions: the indicator equals 1 if a household has a flushable sitting toilet and 0 otherwise, and this proxy is incorporated into rural subsample regressions to capture rural environmental governance.
